# Silencing of Glutathione Peroxidase 3 through DNA Hypermethylation Is Associated with Lymph Node Metastasis in Gastric Carcinomas

**DOI:** 10.1371/journal.pone.0046214

**Published:** 2012-10-10

**Authors:** Dun-Fa Peng, Tian-Ling Hu, Barbara G. Schneider, Zheng Chen, Ze-Kuan Xu, Wael El-Rifai

**Affiliations:** 1 Department of Surgery, Vanderbilt University Medical Center, Nashville, Tennessee, United States of America; 2 Department of Medicine, Vanderbilt University Medical Center, Nashville, Tennessee, United States of America; 3 Department of Cancer Biology, Vanderbilt University Medical Center, Nashville, Tennessee, United States of America; 4 Department of Veterans Affairs Tennessee Valley Healthcare System, Nashville, Tennessee, United States of America; 5 Department of General Surgery, the First Affiliated Hospital of Nanjing Medical University, Nanjing, China; 6 Institute of Tumor Biology, Jiangsu Province Academy of Clinical Medicine, Nanjing, China; Institute of Pathology, Germany

## Abstract

Gastric cancer remains the second leading cause of cancer-related death in the world. *H. pylori* infection, a major risk factor for gastric cancer, generates high levels of reactive oxygen species (ROS). Glutathione peroxidase 3 (GPX3), a plasma GPX member and a major scavenger of ROS, catalyzes the reduction of hydrogen peroxide and lipid peroxides by reduced glutathione. To study the expression and gene regulation of GPX3, we examined GPX3 gene expression in 9 gastric cancer cell lines, 108 primary gastric cancer samples and 45 normal gastric mucosa adjacent to cancers using quantitative real-time RT-PCR. Downregulation or silencing of GPX3 was detected in 8 of 9 cancer cell lines, 83% (90/108) gastric cancers samples, as compared to non-tumor adjacent normal gastric samples (*P*<0.0001). Examination of GPX3 promoter demonstrated DNA hypermethylation (≥10% methylation level determined by Bisulfite Pyrosequencing) in 6 of 9 cancer cell lines and 60% of gastric cancer samples (*P* = 0.007). We also detected a significant loss of DNA copy number of GPX3 in gastric cancers (*P*<0.001). Treatment of SNU1 and MKN28 cells with 5-Aza-2′ Deoxycytidine restored the GPX3 gene expression with a significant demethylation of GPX3 promoter. The downregulation of GPX3 expression and GPX3 promoter hypermethylation were significantly associated with gastric cancer lymph node metastasis (*P* = 0.018 and *P* = 0.029, respectively). We also observed downregulation, DNA copy number losses, and promoter hypermethylation of GPX3 in approximately one-third of tumor-adjacent normal gastric tissue samples, suggesting the presence of a field defect in areas near tumor samples. Reconstitution of GPX3 in AGS cells reduced the capacity of cell migration, as measured by scratch wound healing assay. Taken together, the dysfunction of GPX3 in gastric cancer is mediated by genetic and epigenetic alterations, suggesting impairment of mechanisms that regulate ROS and its possible involvement in gastric tumorigenesis and metastasis.

## Introduction

Gastric cancer (GC) is the fourth most common cancer in the world [1], [2] with about 900,000 new cases diagnosed in the world each year [2]. Gastric cancer remains the second leading cause of cancer-related deaths worldwide [2]. Although there has been a decline in the overall incidence of distal gastric cancer during the past decades, the incidence is rising for adenocarcinomas of the proximal part of the stomach in the Western world [2]. It is generally agreed that gastric cancer is a multifactor disease in which *Helicobacter pylori (H. pylori)* infection plays a crucial role, especially for distal gastric cancer [3]–[5]. Accumulating data indicate that *H. pylori* infection generates high levels of reactive oxygen species (ROS) from multiple sources [6]. Activated neutrophils, for example, are the main source of ROS production in the *H. pylori*-infected stomach; however, *H. pylori* itself can also produce ROS [7]. In addition, extensive recent studies have revealed that *H. pylori*-induced ROS production in gastric epithelial cells might affect gastric epithelial cell signal transduction, resulting in gastric carcinogenesis [8]–[10]. Excessive ROS production in the stomach promotes DNA damage in gastric epithelial cells, suggesting its involvement in gastric carcinogenesis [7], [11], [12].

Normal cells have intact antioxidative properties that protect cells from ROS-induced DNA damage and cell injury [13]–[15]. Among these systems, the glutathione peroxidase family (GPXs) is a major antioxidative enzyme family that catalyzes the reduction of hydrogen peroxide, organic hydroperoxide, and lipid peroxides by reduced glutathione [15]–[19]. Glutathione peroxidase 3 (GPX3), also named plasma glutathione peroxidase, is the only known selenocysteine containing an extracellular antioxidant isoform [20]. The human GPX3 gene is approximately 10 kb in length, spanning 5 exons on chromosome 5q32. [20], [21]. It has been reported that GPX3 catalyzes the reduction of hydrogen peroxide and lipid peroxides and is a major scavenger of ROS produced during normal metabolism or after oxidative insult [22], [23]. GPX3 is selectively expressed in normal human tissues, including the gastrointestinal tract. However, downregulation of GPX3 has been recently reported in multiple human cancers such as prostate, esophageal, and bladder cancer [24]–[26], suggesting its importance in human tumorigenesis. In the present study, we examined GPX3 gene expression, gene promoter methylation status, and copy number in a panel of primary gastric cancers and correlated it with clinicopathological parameters.

## Materials and Methods

### Ethics Statement

De-identified human tissue samples were obtained from the archives of pathology at Vanderbilt University (Nashville, TN, USA) and from the National Cancer Institute Cooperative Human Tissue Network (CHTN). The use of specimens was approved by the Institutional Review Board at Vanderbilt University Medical Center. All patients provided written consent, and samples were collected after surgical resection. All tissue samples that were included in this study were collected from tissues that remained after the completion of diagnosis and are otherwise discarded.

### Tissue samples

All tissue samples were obtained from the pathology archives at Vanderbilt University (Nashville, TN, USA) and from the National Cancer Institute Cooperative Human Tissue Network (CHTN). The use of specimens from the archival tissue repository was approved by the Institutional Review Board. All tissue samples included in this study were coded and collected from tissues that remained after the completion of diagnosis and that were otherwise discarded. Among 108 patients, 70 male and 38 female, ages ranging from 38–87 years of age with a median age of 64 years. All tumors were histologically verified. The gastric adenocarcinomas ranged from well-differentiated (WD) to poorly differentiated (PD), stages I to IV, with a mix of intestinal- and diffuse-type tumors. The “normal" samples in this study were the gastric mucosal epithelial tissues adjacent to the cancers without neoplastic changes.

### Cell lines

Nine gastric cancer cell lines (AGS, MKN28, MKN45, MKN75, KATO3, SNU1, SNU5, SNU16, and RF1) were purchased from American Type Culture Collection (Manassas, VA, http://www.atcc.org) and Riken (Ibaraki, Japan; http://www.brc.riken.go.jp/lab/cell/english). Cells were maintained in either DMEM medium or RPMI 1640 medium with a supplement of 10% fetal bovine serum and antibiotics. All cell lines were cultured at 37°C with 5% CO_2_.

### Quantitative Real-Time Reverse Transcription PCR (qRT-PCR) Analysis of GPX3

Total RNA was isolated using the RNeasy Mini-kit (Qiagen, Valencia, CA, USA). Single-stranded cDNA was subsequently synthesized using the iScript cDNA Synthesis Kit (Bio-Rad, Hercules, CA, USA). Expression of GPX3 was evaluated for a set of 153 frozen primary human samples including 108 samples of gastric carcinoma and 45 samples of normal gastric mucosa adjacent to cancers. For 24 tumors, matching normal mucosa was available from the same patients. The GPX3 primers (forward 5′-GCCGGGGACAAGAGAAGT-3′ and reverse 5′-GAGGACGTATTTGCCAGCAT-3′) were designed using the online software, Primer 3 (http://frodo.wi.mit.edu/). The qRT-PCR was performed using an iCycler (Bio-Rad) with the threshold cycle number determined by use of iCycler software, version 3.0. Reactions were performed in triplicate and the threshold numbers were averaged. Results for the GPX3 gene were normalized to HPRT1 gene (forward 5′-TTGGAAAGGGTGTT TATTCCTCA-3′ and reverse 5′-TCCAGCAGGTCAGC AAAGAA-3′), which had minimal variation in all normal and tumor samples tested, and is therefore considered to be a reliable and stable reference gene for RT-PCR. Expression was calculated by use of the formula 2^(*Rt*–*Et*)^/2^(*Rn*–*En*)^ as previously described [27], [28]. For all of the primary gastric carcinoma samples, the gene was considered downregulated, if the relative mRNA expression was ≤0.5 [29].

### DNA Bisulfite Treatment and Pyrosequencing Analysis

DNA was purified using a DNeasy Tissue Kit (Qiagen). The bisulfite modification of the DNA from cell lines and tissues was performed using an EZ DNA Methylation-Gold Kit (Zymo Research, Orange, CA), according to the manufacturer's protocol. The GPX3 promoter CpG island was identified by using a CpG island online search tool (http://www.uscnorris.com), as previously described [28]. The Pyrosequencing primers were designed using PSQ Assay Design Software (Biotage, Uppsala, Sweden). The forward primer sequence was AGGTGGGGAGTTGAGGGTAA, the reverse biotin-labeled primer sequence was Biotin-TCCCAACCACCTTTCAAAC, and the sequencing primer was GGGAGTTGAGGGTAAGT. A 40 ng aliquot of modified DNA was subjected to polymerase chain reaction (PCR) amplification of the specific promoter region using the above primers and the Platinum PCR SuperMix High Fidelity Enzyme Mix (Invitrogen, Carlsbad, CA). The PCR products were checked by gel electrophoresis to confirm the size of the product and rule out the formation of primer dimers. The specific PCR products were then subjected to quantitative Pyrosequencing analysis using a Biotage PyroMark MD System (Biotage), following the protocol provided by the manufacturer. The results were analyzed by Pyro Q-CpG 1.0.9 software (Biotage). Based on the methylation levels in the normal samples, we used 10% methylation as a cutoff for the identification of DNA hypermethylation of the GPX3 promoter. Statistical analysis was performed to detect significant changes in the frequencies of DNA methylation of the CpG sites between tumor and normal samples.

### 5-Aza-2′ Deoxycytidine and Trichostatin-A Treatment

For validation of the role of promoter DNA hypermethylation in transcriptional regulation of GPX3 *in vitro*, gastric cancer cell lines SNU1 and MKN28 were used. SNU1 and MKN28 cells were maintained in Dulbecco's modified Eagle's medium (DMEM) supplemented with 10% fetal bovine serum (FBS) and antibiotics (Invitrogen). Cells were seeded at low density for 24 hours and then treated with 5 µM 5-Aza-2′ deoxycytidine (5-Aza, Sigma-Aldrich, St. Louis, MO) for 72 hours or 300 nM Trichostatin-A (TSA, Wako, Osaka, Japan) for 24 hours. Total RNA and DNA were isolated and purified by RNeasy and DNeasy Tissue kits (Qiagen), as described above. DNA methylation levels of the CpG nucleotides of the GPX3 promoter were determined by Pyrosequencing. The GPX3 mRNA expression levels were determined by qRT-PCR, as described above.

### Immunofluorescence staining of GPX3 protein in SNU1 cells

To check GPX3 protein expression after 5-Aza treatment, we performed immunofluorescence staining against GPX3 in SNU1. SNU1 cells treated with 5 µM 5-Aza (Sigma-Aldrich) for 72 hours and/or 300 nM Trichostatin-A (TSA, Wako) for 24 hours, fixed with fresh 4% paraformaldehyde for 45 min at room temperature, followed by permeabilization with 0.1% Triton X-100 in 0.1% sodium citrate for 2 min on ice. Cells were then incubated with 10% normal goat serum (Invitrogen) for 20 min at room temperature. Cells were incubated with primary antibody against GPX3 (Rabbit, 1∶500; Abnova, Taiwan) overnight at 4°C followed by secondary goat anti-rabbit antibody conjugated with Alexa Fluor 488 (1∶1000, Invitrogen) at room temperature for 45 min. The slides were mounted using Vectashield with DAPI (Vector Laboratories, Burlingame, California, USA) and viewed under a fluorescence microscope.

### Measurement of DNA Copy Number by Quantitative PCR (qPCR)

For evaluation of relative DNA copy numbers, we performed the qPCR amplifications using an iCycler (Bio-Rad). PCR reactions were prepared in a total volume of 20 µl containing template DNA (40 ng), with the threshold cycle number determined by use of iCycler software version 3.0. Primers were designed using the online software Primer 3 (http://frodo.wi.mit.edu/). The forward and reverse primers for GPX3 genomic DNA were 5′-CCCCTTCAGTAGGGCCTAAG-3′ and 5′-TTCTTCAGGACCAGGACCAC-3′, respectively. The primers were obtained from Integrated DNA Technologies (Coralville, Iowa). Reactions were performed in triplicate, and the threshold numbers (CT) were averaged. The results were normalized to the average CT of both β-Actin and GAPDH, which generated similar results and had minimal variation in all normal and tumor samples tested. DNA copy number was calculated in the same way as the quantification of qRT-PCR for mRNA expression [29] and normalized to the average level of 10 blood DNA samples of normal individuals in which the GPX3 copy number are not affected ( 2 copies, equal to copy number ratio 1.0). Loss of DNA copy number was considered at a relative cutoff ratio ≤0.5.

### Construction of GPX3 expression adenoviral system

The full length of GPX3 coding sequence with Flag-tag was amplified from normal cDNA by PCR using Platinum PCR SuperMix High Fidelity (Invitrogen) and was cloned into the pACCMV.pLpA plasmid. The pACCMV.pLpA-GPX3 plasmid was then co-transfected with pJM17 plasmid into 293 AD cells to generate and propagate the recombinant GPX3-expressing adenoviral particles as previously described [30]. The viruses were plaque purified, and the titer of the virus was determined using the Adeno-X qPCR Titration Kit (Clontech, California, USA) following the manufacturer's instructions.

### Colony formation assay

AGS cells were infected with 25 MOI control or GPX3-expressing adenoviral particles. 48 hours after infection, cells were split and seeded (500 cells/well) in 6-well plates. Cells were cultured at 37°C for another 2 weeks. Cells then were stained with 0.05% crystal violet. The images of the plates were analyzed using Image J software. Each experiment was set in triplicate and statistical analysis was done using Prism software. The relative number of colonies in GPX3-expressing cells was adjusted to control cells.

### Colony formation in soft agar

To check if GPX3 was also involved in anchorage-independent growth, soft agar colony formation assay was performed. In brief, AGS and MKN28 cells were infected with 25 MOI control or GPX3-expressing adenoviral particles. 48 hours after infection, cells were split and seeded (1.0×10^5^ cells/well) in a 0.35% noble agar (Sigma) mixed with culture medium (on the top of 0.5% noble agar with medium) in 6-well plates. Cells were cultured at 37°C for another 2 weeks. The images of the plates were captured under a microscope and analyzed using Image J software. Each experiment was set in triplicate and statistical analysis was done using Prism software. The relative number of colonies in GPX3-expressing cells was adjusted to control cells.

### Scratch wound healing assay

To check if GPX3 has a role in cell migration, we performed a scratch wound healing assay in AGS cells [31]. AGS cells were infected with 25 MOI GPX3-expressing or control adenoviruses. When cells were 100% confluent, a scratch was made across the plates using a pipette's tip. Cells were cultured in complete medium and images were taken every 24 hours to monitor the wound healing process.

### Statistical Analysis

GraphPad Prism software version 4.0 (GraphPad Prism Software, La Jolla, CA) was used for all of the statistical analyses. The Student *t* test was used to compare the DNA methylation level and mRNA ratios between normal and gastric cancers in matched samples (paired t-test) and unmatched samples (unpaired t-test). In addition, we analyzed the association between DNA methylation and clinicopathological factors. The correlation between the DNA methylation level and mRNA expression was determined by Spearman's rank correlation. The comparison of GPX3 gene expression or DNA methylation level with clinicopathological factors was made by Chi-square/Fisher's exact tests or unpaired t-test. All *P* values were based on two-tailed tests and differences were considered statistically significant when the *P* value was ≤.05.

## Results

### GPX3 was frequently downregulated or silenced in gastric carcinomas

**Figure 1:**
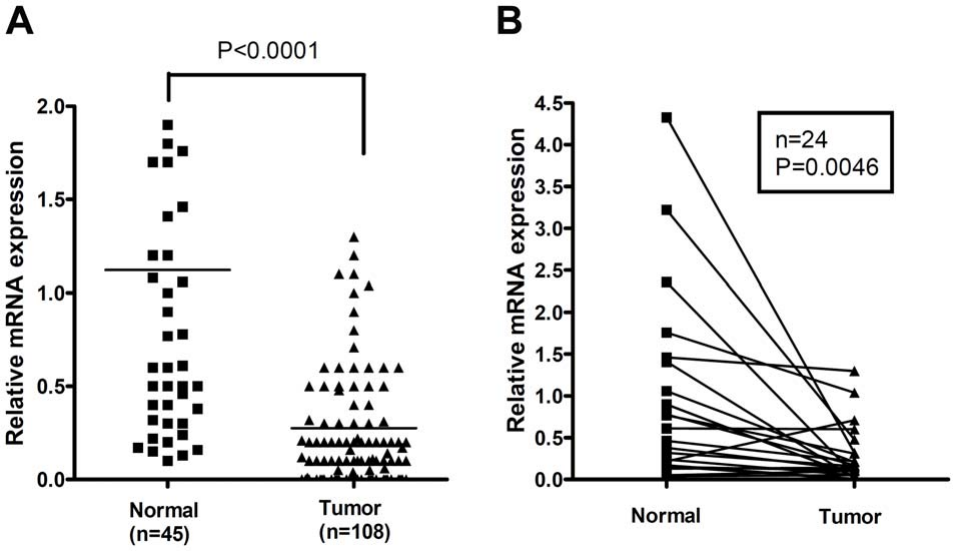
GPX3 is downregulated in gastric cancers. 108 gastric cancer samples and 45 tumor-adjacent histologically normal gastric mucosa samples were analyzed by real-time RT-PCR for GPX3 expression, which was significantly downregulated in gastric cancers as compared to adjacent normal samples (A). A similar result was confirmed in 24 matched normal and tumor samples from the same patients (B).

The qRT-PCR analysis demonstrated that GPX3 mRNA expression was frequently downregulated in gastric cancer cell lines (8/9 cell lines examined, Table 1) and in primary gastric cancer tissue samples (90/108, 83%) as compared to 45 normal samples (*P*<.0001; Figure 1A). Further analysis of 24 paired tumor and normal samples confirmed the significant downregulation of GPX3 mRNA expression in tumors as compared with their corresponding normal samples (Figure 1B). Moreover, about one-third (36/108, 33%) of primary gastric cancers and 6/9 of the cancer cell lines (Table 1) showed complete silencing of GPX3 mRNA expression, as indicated by the absence of a detectable signal. Notably, about 33% (15/45) of the “normal" gastric tissues adjacent to cancers without neoplastic changes also displayed downregulation (relative expression, ≤0.5) of GPX3 mRNA, as normalized to the value of the average of all normal samples.

**Table 1:** DNA copy number, methylation level and gene expression of GPX3 in gastric cancer cell lines.

Sample	DNA Copy number ratio	% Methylation	mRNA expression ratio
Normal	1.0	5	1
AGS	0.5	3	0.1
MKN28	0.6	91	0
MKN45	0.3	92	0
MKN75	0.6	91	0
KATO3	0.2	88	0
SNU1	0.4	94	0
SNU5	0.8	4	0.9
SNU16	0.3	6	0.1
RF1	0.6	24	0

Copy number of the GPX3 gene was determined using qPCR and normalized to 10 normal blood DNA samples as described in the Methods section. The real copy number of each sample equals to the above copy number ratio times 2. DNA methylation levels were determined using Pyrosequencing and are shown as an average level of the 8 CpG sites in the GPX3 promoter region. GPX3 mRNA expression fold was determined using real-time RT-PCR and normalized to the average value of normal gastric samples as described in the Methods section.

### Promoter DNA hypermethylation of the GPX3 gene correlates with downregulation of mRNA expression

**Figure 2:**
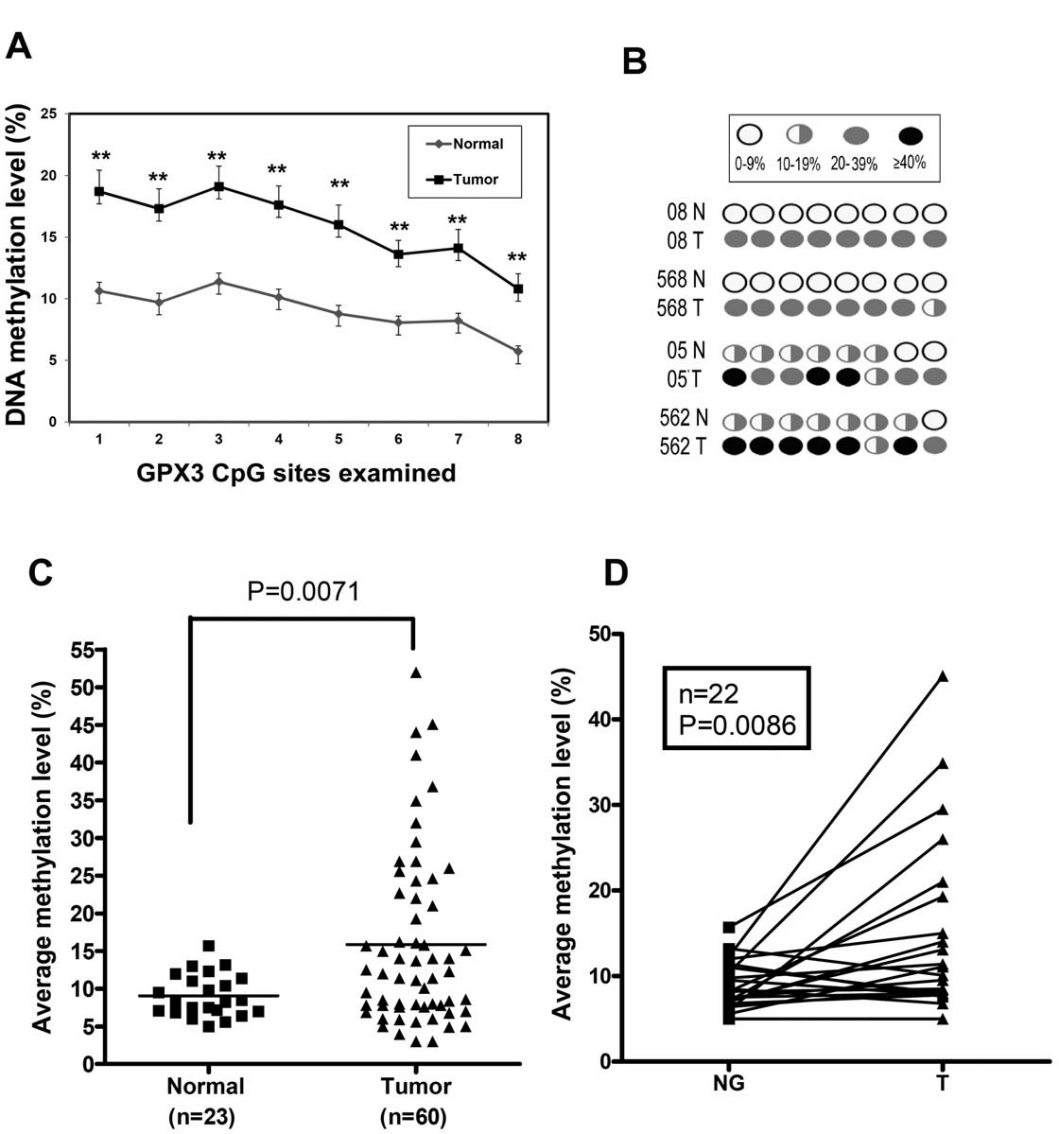
GPX3 promoter was hypermethylated in gastric cancers. DNA methylation level of 8 CpG sites in the GPX3 promoter was quantitated by Pyrosequencing. (A) A schematic profile of GPX3 methylation of the 8 CpG sites was examined in 4 matched normal (N) and tumor (T) samples. (B) The DNA methylation level of each of the 8 CpG sites in 23 normal samples and 60 tumor samples were measured. * *P*<0.01. (C) The average DNA methylation level of the 8 CpG sites in 23 normal samples and 60 tumor samples is shown. (D) The average DNA methylation level of the 8 CpG sites in 22 matched normal and tumor samples from the same patients is indicated.

**Figure 3:**
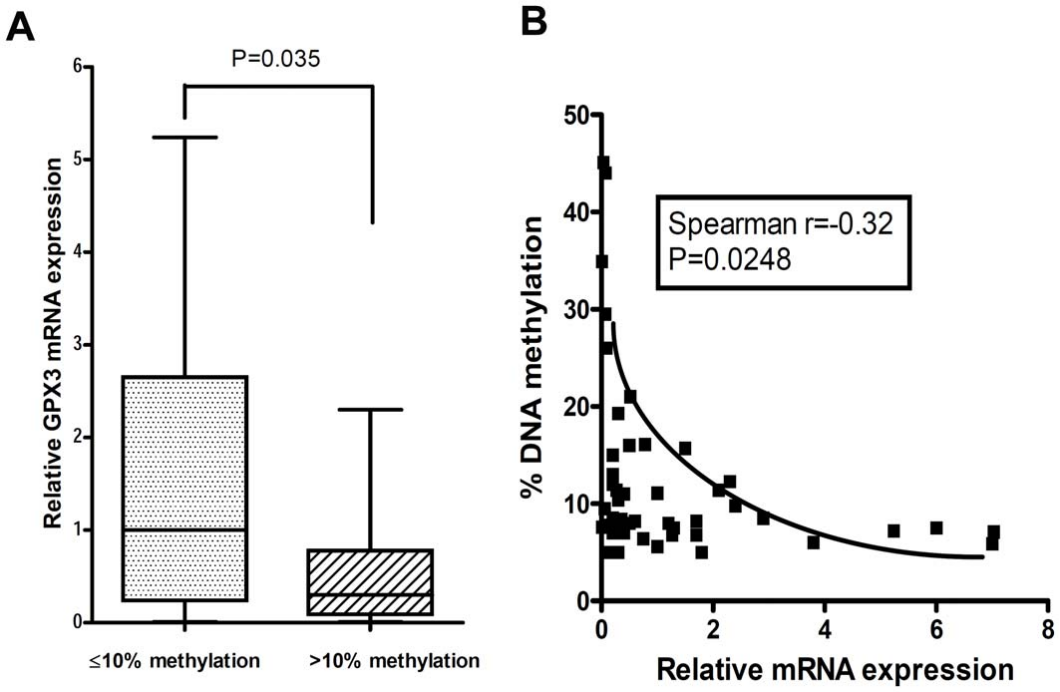
GPX3 promoter methylation level inversely correlates with GPX3 gene expression. (A) GPX3 expression level in gastric cancers with GPX3 hypermethylation (≥10%) was significantly lower than that in gastric cancers without hypermethylation (<10%). (B) Spearman analysis of all normal and tumor samples with GPX3 gene expression and DNA methylation demonstrated a significant inverse correlation(r = −0.32) between DNA hypermethylation and gene expression.

The DNA methylation changes, which were determined by Pyrosequencing technology, were not associated with the patients' ages. A representative DNA methylation scheme of each CpG sites in the GPX3 promoter of 4 matched normal and tumors is shown in Figure 2A. Quantitative analysis of GPX3 promoter DNA methylation, indicated increased promoter DNA methylation levels of all tested CpG nucleotides in tumor samples compared with normal samples (Figure 2B). The average methylation level for the GPX3 gene promoter in gastric cancers was significantly higher than that in normal samples (*P* = 0.007, Figure 2C). Hypermethylation of the GPX3 promoter (≥10%) was detected in 60% (36/60) of the gastric cancers and 6/9 cancer cell lines (Table 1), consistent with our results showing GPX3 downregulation. We observed that 39% (9/23) of the normal gastric tissues adjacent to cancers showed an increased DNA methylation level of the GPX3 promoter from 10% to 15%. Analysis of DNA methylation in 22 tumor samples and their matching tumor-adjacent normal gastric mucosae demonstrated a similar significant increase in the level of DNA methylation in tumors compared with their controls (*P* = 0.0086, Figure 2D). We next analyzed the promoter DNA methylation against mRNA expression levels in all samples. As shown in Figure 3A, samples with hypermethylation (>10%) had significantly lower levels of GPX3 expression (*P* = 0.035) compared with samples with absent or low promoter methylation levels (≤10%). Using the Spearman rank correlation, we found a significant inverse correlation between promoter methylation and mRNA expression of GPX3 (coefficient *r* = −0.32, *P* = 0.024; Figure 3B). These results suggest that the hypermethylation of the GPX3 promoter region is one of the factors involved in the suppression of its mRNA expression in gastric cancers.

### 5-Aza-2′ Deoxycytidine (5-Aza) and Trichostatin-A (TSA) treatment restored GPX3 expression in silenced gastric cancer cell lines

**Figure 4:**
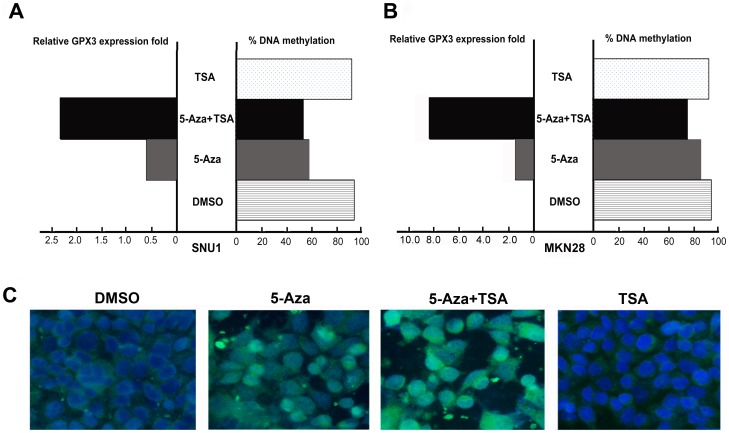
5-Aza treatment restored GPX3 gene expression in gastric cancer cell lines with silenced GPX3. SNU1 and MKN28 cancer cells were treated with 5-Aza and TSA as described in the Methods section. In A (SNU1 cells) and B (MKN28 cells), relative GPX3 expression ratios normalized to HPRT are shown in the left panel. DNA methylation levels of corresponding samples are shown on the right panel. C shows immunofluorescence staining of GPX3 protein in SNU1 cells. 5-Aza, 5-Aza-2′ deoxycytidine. TSA, Trichostatin-A.

As shown in Figure 4, the 5-Aza treatment of the SNU1 (A) and MKN28 (B) cell lines with its fully methylated GPX3 promoter (Table 1) restored GPX3 mRNA expression, and this restoration of gene expression was associated with promoter demethylation. TSA treatment alone had no effect in restoring the GPX3 expression or in altering the methylation levels. However, administration of TSA following 5-Aza had a significant additive effect in restoring gene expression. Furthermore, TSA treatment following 5-Aza led to additional gene expression with a further decrease in the methylation level of GPX3. To check if 5-Aza treatment also restored GPX3 protein level, we performed an immunofluorescence assay using antibody against GPX3 in SNU1 cells. As shown in Figure 4C, there was a significant increase in the GPX3 green immunofluorescence signal after 5-Aza and 5-Aza-TSA treatments as compared to DMSO control, suggesting that 5-Aza and 5-Aza-TSA can restore the GPX3 protein expression in these cells.

### Loss of DNA copy numbers cooperates with DNA hypermethylation for silencing GPX3 mRNA expression in gastric cancer

**Figure 5:**
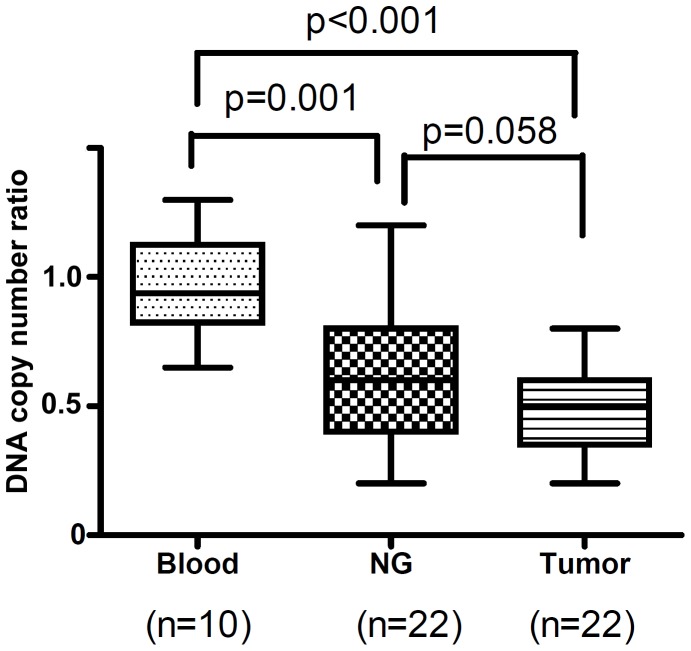
GPX3 copy number loss in gastric cancers. GPX3 copy number was determined by qPCR and normalized to both beta-actin and GAPDH of the same samples. The results were then compared to 10 blood DNA samples from normal individuals in which GPX3 copy number was assumed to be 2.0 (copy number ratio equals to 1.0 in Table 1). Loss of GPX3 copy number was detected in both gastric cancer samples and adjacent normal samples.

Although GPX3 promoter hypermethylation correlated statistically with low gene expression levels, we detected silencing of GPX3 mRNA expression in 83% (90/108) of gastric cancers, whereas promoter hypermethylation was seen in only 60% (36/60) of gastric cancers. These findings prompted us to find out whether loss of copy numbers could be a contributing factor in silencing GPX3 expression. Evaluation of relative DNA copy numbers of the 9 gastric cancer cell lines clearly demonstrated that 5 of the 9 cell lines have lost GPX3 copy number (copy number ratio≤0.5) as compared to the normal cells, which have a copy number of 2 (Table 1). In particular, in AGS and SNU16, loss of DNA copy number seems to account for GPX3 downregulation. While in the other 6 cell lines (MKN28, MKN45, MKN75, KATO3, SNU1, and RF1), both loss of copy number and DNA hypermethylation are associated with the silencing. The only cell line (SNU5) lacking both DNA hypermethylation and copy number loss also expresses GPX3 similar to the level in normal samples. The DNA copy number of twenty-two gastric cancer samples and 22 tumor-adjacent “normal" stomach samples were compared with the average copy number of 10 blood samples from normal individuals. The GPX3 copy numbers in the 10 blood samples were used as an accurate reference for calculating the ratio and normalization in gastric tissues. As shown in Figure 5, there was a significantly lower level of GPX3 DNA copy numbers in the gastric cancers and the tumor-adjacent “normal" stomach tissue samples, as compared to that in normal blood samples (*P* = 0.001). The comparison of GPX3 copy number in 22 matched tumor and normal samples demonstrated lower levels of DNA copy numbers in tumors (*P* = 0.058).

### Loss of GPX3 correlated with tumor lymph node metastasis

**Figure 6:**
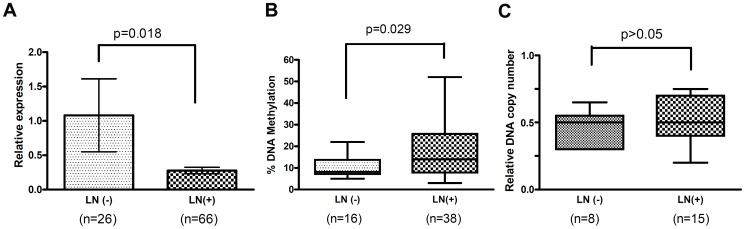
Dysfunction of GPX3 correlated with lymph node metastasis. (A) Loss of GPX3 gene expression correlated with lymph node metastasis. (B) Increase in GPX3 methylation correlated with lymph node metastasis. (C) GPX3 copy number change did not correlate with lymph node metastasis.

Based on the available clinicopathological information, we found both GPX3 hypermethylation and downregulation of GPX3 expression significantly correlated with lymph node metastasis (*P* = 0.029 and *P* = 0.018 for DNA methylation and gene expression, respectively; Figure 6). No significant correlation was found between either DNA methylation or gene expression, and other parameters examined, such as patient age, sex, tumor grade, and invasion depth. GPX3 DNA copy number did not correlate with lymph node metastasis and other parameters, possibly due to the small number of samples.

### Reconstitution of GPX3 in gastric cancer cells did not suppress tumor cell growth but inhibited tumor cell migration

**Figure 7:**
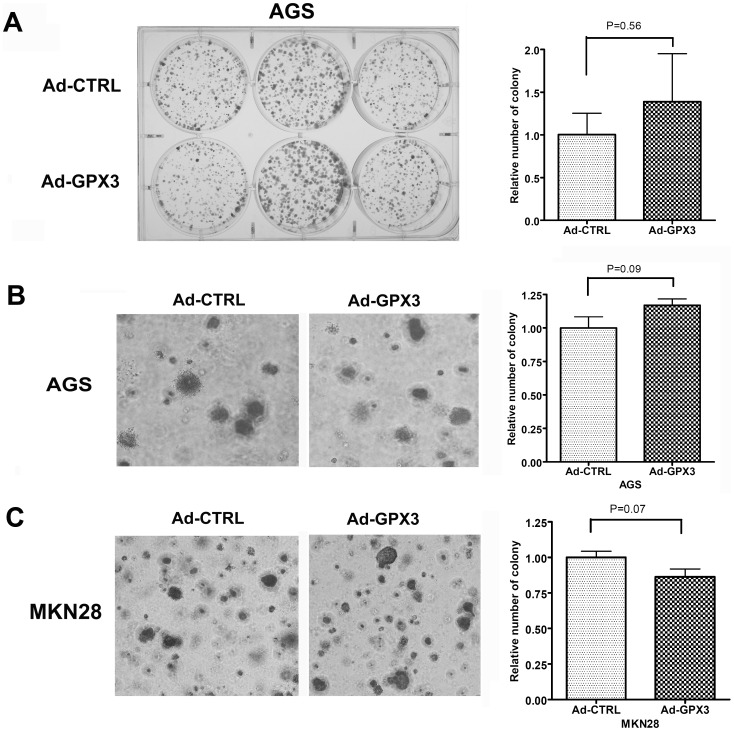
Reconstitution of GPX3 did not suppress tumor cells growth. (A) Colony formation assay in AGS cells. (B) Soft agar colony formation assay in AGS cells. (C) Soft agar colony formation assay in MKN28 cells. Right panels in A, B and C demonstrate the quantitative data using Image J software.

**Figure 8:**
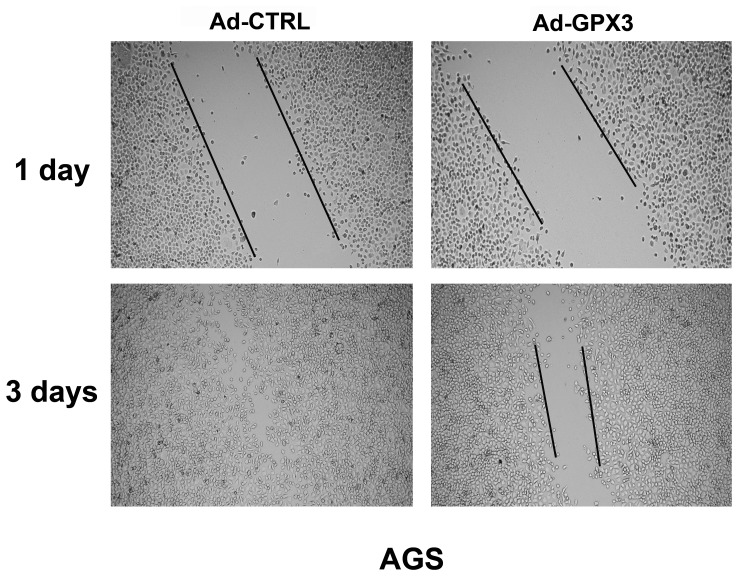
Reconstitution of GPX3 expression suppressed tumor cell migration. AGS cells were infected with Ad-GPX3 and Ad-CTRL. When cells were confluent (48 hours after viral infection), a scratch wound healing assay was performed. The scratch gap was periodically monitored and recorded.

Because GPX3 has been suggested to be a potential tumor suppressor in prostate cancer [26], we hypothesized that GPX3 may have the similar tumor suppressor function in gastric cancer. We reconstituted GPX3 gene expression in AGS and MKN28 cell lines and performed colony formation and soft agar colony formation assays. Interestingly, we did not observe any significant difference in the number of colonies between GPX3-expressing AGS cells and the control cells (Figure 7A). Similar results were obtained by soft agar assay in AGS cells and MKN28 cells (Figures 7B&7C). In contrast, scratch wound healing assay data indicated that GPX3-expressing AGS cells exhibited significantly slower repair of the scratched wound as compared to control cells, suggesting that GPX3 expression could impair cell migration (Figure 8).

## Discussion

The glutathione peroxidase family (GPXs) is a major antioxidative enzyme family that catalyzes the reduction of hydrogen peroxide, organic hydroperoxide, and lipid peroxides [15]–[19]. In this process, GPXs convert oxygen superoxide and hydrogen peroxide, a major ROS which have been reported to induce oxidative DNA damage [30], to harmless intermediate products, and therefore play critical roles in protecting cells from DNA damage. GPX3 is a secreted form of the GPX family that is readily detectable in plasma and mucosal surfaces, and detoxifies ROS before it can enter into cells [17]. *H. pylori* infection is the main risk factor for gastric tumorigenesis which leads to pro-inflammatory response and generation of high levels of ROS in stomach with a significant increase in oxidative DNA damage [7]. ROS-induced DNA damage leading to disruption of genomic integrity has been shown to be an important cause of human cancers [14]. The loss of GPX-mediated activities may be associated with early stages of inflammation-mediated carcinogenesis [16]. Therefore, our results showing frequent loss of GPX3 expression in gastric cancer may underscore the failure in the cellular antioxidant system which is the first line of defense against detrimental ROS activity.

Downregulation of GPX3 has been reported in human cancers such as lung, ovarian, bladder, esophageal, and prostate cancer [24]–[26], [28], [32], as well as in gastric cancers [33], [34]. Mono-allelic hypermethylation and inactivation of GPX3 in benign precursor lesions, metaplasia, and dysplasia of the esophagus has been reported; while inactivation of both alleles was detected in invasive carcinoma [25]. Complete inactivation of the GPX3 gene by genetic loss of one allele and methylation-mediated silencing of the remaining allele was also reported in prostate cancer [26]. In the current study, we have demonstrated promoter hypermethylation and DNA copy number loss as possible mechanisms mediating silencing of GPX3 in gastric cancers, although other mechanisms such as gene mutation and miRNA regulation may also be involved and need to be further studied. The occurrence of both genetic and epigenetic alterations in GPX3 suggests that silencing of this gene could be a critical event in the multi-step gastric tumorigenesis cascade. Of note, we observed mRNA downregulation, promoter hypermethylation and DNA copy number loss of GPX3 in a number of tumor-adjacent “normal" gastric mucosae samples. These tumor-adjacent “normal" tissues, although histologically normal, they usually have some degree of inflammation and could possibly have changes at the molecular level. Therefore, our data suggest that silencing of GPX3 is possibly an early event in gastric tumorigenesis. Given the known function of GPX3, its downregulation is expected to result in impairment in the antioxidant capacity of cells with possible accumulation of oxidative DNA damage at the early stage of gastric tumorigenesis. Further studies on GPX3 methylation, inflammation and *H. pylori* infection are needed to fully elucidate the relationship among them.

A recent study has demonstrated that GPX3 can suppress prostate cancer growth and metastasis [26]. Unfortunately, we did not confirm the similar tumor suppression role of GPX3 in two gastric cancer cell lines (AGS and MKN28), suggesting that GPX3 may have differential functional roles in different organs. Nonetheless, our scratch wound healing assay data indicated that GPX3-expressing cells have impairment in their migration capacity. This is in accordance with our findings showing a significant correlation of GPX3 downregulation and hypermethylation with lymph node metastasis. Further studies are necessary to confirm this finding using alternative assays and to elucidate the underlying mechanisms of how GPX3 potentially regulates cancer metastasis.

In conclusion, our results suggest that GPX3 gene inactivation by promoter methylation and/or copy number loss is a frequent finding in gastric cancer that correlates with increased incidence of lymph node metastasis. This loss may account for a reduced antioxidant capacity and accumulation of oxidative DNA damage observed in gastric tumorigenesis. Further studies to explore the functions of GPX3 in gastric tumorigenesis are required to address its potential role in the development and progression of gastric cancer, in particular, its potential roles in tumor cell migration and metastasis.
